# Organised crime and the efforts to combat it: a concern for public health

**DOI:** 10.1186/1744-8603-6-21

**Published:** 2010-11-15

**Authors:** Lucy Reynolds, Martin McKee

**Affiliations:** 1 Centre for Research on Drugs and Health Behaviour, Social and Environmental Health Research Department, Faculty of Public Health Policy, London School of Hygiene and Tropical Medicine, 15-17 Tavistock Place, London, WC1 H 9SH, UK; 2 European Centre on Health of Societies in Transition, Faculty of Public Health Policy, London School of Hygiene and Tropical Medicine, 15-17 Tavistock Place, London, WC1 H 9SH, UK

## Abstract

This paper considers the public health impacts of the income-generating activities of organised crime. These range from the traditional vice activities of running prostitution and supplying narcotics, to the newer growth areas of human trafficking in its various forms, from international supply of young people and children as sex workers through deceit, coercion or purchase from family, through to smuggling of migrants, forced labour and the theft of human tissues for transplant, and the sale of fake medications, foodstuffs and beverages, cigarettes and other counterfeit manufactures. It looks at the effect of globalisation on integrating supply chains from poorly-regulated and impoverished source regions through to their distant markets, often via disparate groups of organised criminals who have linked across their traditional territories for mutual benefit and enhanced profit, with both traditional and newly-created linkages between production, distribution and retail functions of cooperating criminal networks from different cultures. It discusses the interactions between criminals and the structures of the state which enable illegal and socially undesirable activities to proceed on a massive scale through corruption and subversion of regulatory mechanisms. It argues that conventional approaches to tackling organised crime often have deleterious consequences for public health, and calls for an evidence-based approach with a focus on outcomes rather than ideology.

## Introduction

The conceptualisation of health determinants has expanded greatly over recent decades, moving upstream from the causes of ill health, such as smoking or obesity, to the "causes of the causes" [1]. This has been accompanied by a recognition of the health consequences of policies in other sectors, now cast as "Health in all policies"[2] and operationalised in health impact assessment [3]. These changes have led to a burgeoning literature on the health consequences of upstream determinants such as transport, environmental, and agricultural policies. This broader view has paid too little attention to one important area of government policy, that relating to income generation by criminal organisations. What attention has been paid has, rightly, addressed the alleviation of suffering among those affected but it has yet to move upstream to address the phenomenon of organised crime and the disease burden it generates.

Furthermore, where the public health community has become engaged, it has been by groups working in silos isolated from each other, such as those concerned about the victims of trafficking or the effects of cigarette smuggling, but not addressing the fact that many of those these apparently disparate activities are controlled by the same people. We contend that organised crime is a neglected contributor to avoidable ill health that deserves greater attention from public health professionals.

## What is organised crime?

Organized crime was characterised, in 1994, as:

*"group organization to commit crime; hierarchical links or personal relationships which permit leaders to control the group: violence, intimidation and corruption used to earn profits or control territories or markets; laundering of illicit proceeds both in furtherance of criminal activity and to infiltrate the legitimate economy; the potential for expansion into any new activities and beyond national borders; and cooperation with other organized transnational criminal groups." *[4]

It is increasingly global. Although links between, for example, mafia groups in Italy and the USA have existed for decades, new and rapid means of communication have facilitated the development of international networks. Some build on shared linguistic or cultural ties, such as a network trafficking drugs and human organs, which links criminal gangs in Mozambique, Portugal, Brazil, Pakistan, Dubai and South Africa [5]. Others bring together much less likely groups, such as those trafficking arms, drugs and people between South Africa, Nigeria, Pakistan and Russia, or those linking the Russian mafia with Columbian cocaine cartels or North American criminal gangs with the Japanese Yakuza. Trafficked commodities may pass from group to group along the supply chain; for instance heroin in Italy has traditionally been produced in Afghanistan, transported by Turks, distributed by Albanians, and sold by Italians [6].

Organised crime exploits profit opportunities wherever they arise. Globalisation of financial markets, with free movement of goods and capital, has facilitated smuggling of counterfeit goods (in part a reflection of the creation of global brands), internet fraud, and money-laundering. On the other hand, organised crime also takes advantage of the barriers to free movement of people across national borders and the laws against non-medicinal use of narcotics: accordingly it earns vast profits in smuggling migrants and psychoactive drugs. Briquet and Favarel have identified deregulation and the *"rolling back of the state" *in some countries as creating lacunae that have been occupied by profiteers [7]. The political changes in Europe in the late 1980 s fuelled the growth in criminal networks, often involving former law enforcement officers[8]. Failed states, such as the Democratic Republic of Congo or Sierra Leone, have provided further opportunities as criminal gangs smuggle arms in and commodities out, for example diamonds, gold, and rare earth metals, often generating violence against those involved in the trade and in the surrounding communities [5]. Finally, there are a few states, such as the Democratic Republic of Korea and Burma [5] and Guinea-Bissau (once described as a narco-state [9]) where politicians have been alleged to have played an active role in international crime [6].

Organised criminal gangs have strong incentives. Compared with legitimate producers, they have lower costs of production due to the ability to disregard quality and safety standards, tax obligations, minimum wages or employee benefits. Once established, they may threaten or use violence to eliminate competitors, and can obtain favourable treatment by regulatory authorities either through bribes or threats [5,10].

There is increasing inter-linkage between different types of criminal activity, with distribution channels transporting multiple illegal commodities [11]. Small craft taking cocaine from Colombia return laden with cigarettes from Aruba[12]. Networks developed for arms distribution are being used to traffic in women and drugs[5] while child pornography produced by a notorious Belgian paedophile was distributed along routes set up for international transport of stolen cars and illegal drugs[13].

## Public health implications of organised crime

Organised crime impacts on public health in several ways. One is that it undermines the rule of law. Many public health measures depend, for their effectiveness, on the enforcement of laws, regulations and taxes. Criminal gangs encourage a climate in which officials expect to be bribed, thus undermining enforcement of safety regulations as well as slowing economic growth and prosperity [14]. In some countries recruits to the police force pay an unofficial fee to join which they will subsequently recoup from bribes [15-17].

As a consequence, activities such as traffic enforcement shift from promoting safety to revenue generation. It may be cheaper for food outlets to bribe public health officials rather than keep premises hygienic. However, organised crime also engages in activities that are directly and specifically detrimental to public health, typically involving the production and sale of substandard fake legal goods and illegal drugs, and the trafficking and smuggling of people. We now examine these activities in turn.

## Dangerous products

### Pharmaceuticals

The victims of trade in counterfeit medicines were illustrated graphically in Graham Greene's novel The Third Man, in which Harry Lime diluted stolen penicillin [18]. Lime's successors use the internet, or street markets, rather than the sewers of Vienna to conduct their trade [19] Some counterfeit drugs contain inadequate amounts (or none) of active ingredient, leading to treatment failures or, in the case of anti-microbial agents, resistance: Counterfeit drugs are believed to play a key role in the emergence of resistance to anti-malarials in South East Asia [20]. Products can instead contain too much active ingredient, as has been reported with counterfeit corticosteroids and oestrogens, posing a risk of over-dosage [21]. Other counterfeits are identical to the proprietary versions, except that their provenance is faked so that they are believed by buyers to be something they are not, which contravenes the intellectual property rights of the patent-holder[22]; such merchandise poses no threat to health except insofar as it is supplied to patients without medical supervision. Generic preparations (produced outside patent) should not be confused with counterfeits, but patent-holder lobby groups sometimes encourage such confusion in the interests of reducing their loss of market-share to generic production.

Pharmaceuticals are subject to stringent intellectual property protection. Thus, counterfeiters gain a substantial economic benefit as they are spared the major cost of development and licensing of the products (there is, of course, a separate debate on whether existing intellectual property regimes are themselves beneficial or harmful for public health: the loss of competition from generics enforced by intellectual property laws facilitates elevation of prices of patent-protected drugs, and this price-protection often denies access to essential medicines to those in need). Producers of counterfeits also benefit by substituting high-cost ingredients and ignoring quality control processes, environmental and employee protection, and taxation.

These processes have become extremely sophisticated. One fake medication network involves imports from China to Europe, through the port of Naples, where the local Camorra mafia duplicate the legitimate barcodes on merchandise legally imported into Italy [23]. These are then transferred into a network of unmarked buildings around the port before being fed into official distribution systems[24]

**Figure 1:**
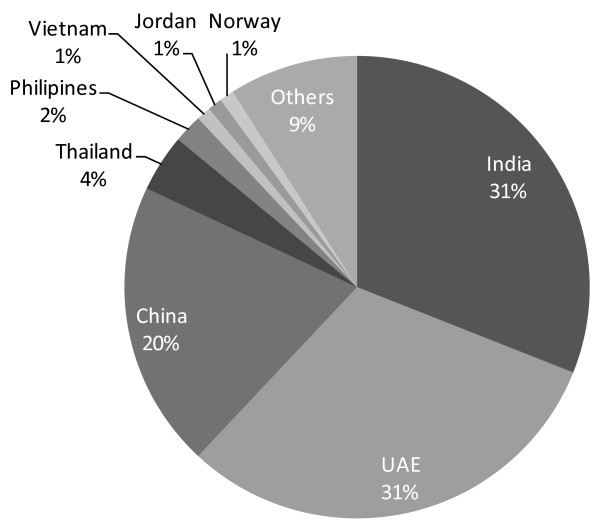
**Country of origin of drugs seized in the European Union on grounds of contravention of intellectual property rights (2006)**. Source: European Commission [117].

The Russian mafiya, Mexican gangs, Chinese triads and Columbian drug cartels have all moved into this form of income generation, a shift that has been attributed to the pressure exerted by the American war on drugs [25]. Consequently, European Customs seizures of medication for contravening intellectual property rights have been increasing steadily. Over three-quarters of seizures in Europe are from three source countries (Figure 1).

By its nature, the global scale of the problem of sale of inactive or dangerous substitutes for medications by criminal organisations is difficult to assess; 115 countries identified counterfeit and/or fake drugs on their markets in 2008 [26]. Some caution is required in interpreting reported data as the global pharmaceutical companies have an interest in portraying some legitimate generics as counterfeit.

The WHO believes that in industrialized countries that operate effective regulatory systems, counterfeit drugs comprise less than 1% of market share, although there have been some important exceptions. For instance, £500,000 worth of Chinese-made counterfeit medicines were discovered in the north of England in early 2009 [27]. Some countries, such as Finland with its long land border with Russia, are especially vulnerable, though much of its counterfeit product is in transit from India or China to Central Europe [28].

The situation is very different in many low- and middle-income countries. Thus, a WHO survey estimated that 30% of drugs sold in Kenya were counterfeit [29] while researchers in Senegal, writing in 2002, found that 21 out of 22 samples of Ampicillin from different suppliers contained only flour [30]. Influenza vaccines consisting only of water were discovered in the Philippines [31] and 2,500 deaths from meningitis followed an immunisation campaign in Niger in which 60,000 people were given a *"vaccine" *composed of nothing but saline [32].

Anti-malarials are among the most frequently counterfeited drugs, with fakes accounting for up to two-thirds of products on sale in some places [33]. For example, *"Coartem tablets" *lacking any active ingredient were found in Ghana in 2009 [34] and it has been estimated that 68% of artesunate purchased in Laos, Burma, Vietnam and Cambodia contained less than half the correct amount of active ingredient [33]. However, the worst situation for public health occurs when traces of the correct ingredient are included in anti-malarials to mislead quality checks relying on colorimetric analysis; such products fuel resistance by supplying a non-therapeutic dose to people being treated for malaria, thus selecting resistant strains. Fake anti-retrovirals have also been identified, in the Ivory Coast [35] and the Democratic Republic of Congo [36], but it is suspected that a systematic investigation would be likely to identify many more examples [37].

Substitution of other active ingredients, with different actions, may occur, such as sulphonamides substituted for the antimalarial halofantrine, metamizole masquerading as artesunate [33], or diazepam replacing cotrimoxazole [22].

### Food and beverages

Illegal trade in food and beverages is less important than in pharmaceuticals because the profit margins are much lower. The problem is mainly concentrated in high value goods, especially alcohol which is subject to excise duties. In some cases, the distinction between legal and illegal trade is hard to define, especially where alcohol ostensibly produced for other purposes is diverted for consumption. Thus, the drinking of aftershaves and industrial spirits has played a major role in the high death toll from alcohol in the former Soviet Union [38]. These products are cheap, as they avoid duty, and highly concentrated, at up to 95% ethanol [39]. Illegal alcohols may contain toxins, typically due to contamination by methanol through the action of pectinase in fruit-based liquor [40], or from addition of industrial methanol. In 2003 counterfeit Johnny Walker Black Label whiskey contaminated with methanol was discovered in Berkshire, England[41]. However, criminal adulteration of foodstuffs can also occur with low-cost products, such as the use of melamine in China to give powdered milk products the appearance of normal protein content (despite a 94% deficit), with fatal consequences for some children to whom it was given [42].

The food supply chain provides other opportunities for organised crime, albeit with less direct health consequences. One example is the use of fraudulent accounting for money laundering of the profits of organised crime, which exploits agricultural subsidies, leading the European Commission to use satellite imaging to check on the existence of olive groves[43]. Another is the mislabelling of foodstuffs, typically concealing their true origins, especially where this confers higher prices. These activities are often linked to, and help to fund, other illegal activities such as bribery and extortion, which may impact more directly on public health.

### Cigarettes

Criminal involvement in the tobacco trade takes two forms. One is the production of counterfeit cigarettes, packaged to look like popular brands. Many of these have been traced to large factories in China [44]. They have been found to contain high levels of poisons such as arsenic and toxic alkaloids, with five times the normal level of cadmium[45], although given the dangers associated with smoking even legally produced cigarettes, this is relatively unimportant in public health terms. Their cheapness compared to licit supplies increases their markets, particularly including accessibility to children.

Much more important is smuggling of cigarettes, enabling them to circumvent the fiscal measures implemented by many governments as an explicit part of their tobacco control strategies, which aim to raise price as a way of restricting demand among less affluent consumers, in particular children. There is a documented pathway for US cigarettes smuggled into Europe: following the last Balkan war, they were loaded into planes in Rotterdam or Switzerland, and unloaded on an unscheduled stop in Montenegro from where they were transported by speedboat to Bari in Italy to the Italian mafia who distributed them. This route yielded cigarettes sold in North London at 30-40% of the price of the same brands bearing tax [5].

As noted above, smuggled cigarettes often travel along the same routes as other contraband, such as narcotics, and there is now substantial evidence that the international tobacco companies have been, at the least, complicit, by failing to take action to reduce leakage of their products into the DNP (duty not paid) market or to employ methods of tracking stock, such as bar coding. At worst they establish a commercial presence in countries as cover for smuggling[46,47] or even build commercial links with smugglers [48-50]. Cheap cigarettes are important in building markets by establishing addiction in the young and in poorer communities, with substantial evidence that major tobacco companies facilitate diversion of supplies into illicit channels for this purpose [51]. As noted above, those involved in cigarette smuggling, some with links to the tobacco industry, may be involved in illegal trade in other goods.

### Other products

Criminal activities have been associated with contamination of a range of other products. For example, fake Colgate toothpaste containing ethylene glycol was identified in dollar stores in several eastern states of the USA in 2007, apparently produced in South Africa [21]. The same chemical was substituted for glycerine in cough medicine imported from China via Spain: it killed more than a hundred Panamanian children in 2006 [52], while its inclusion in a teething product was linked to the deaths of at least 118 Nigerian children in 2008 and 2009 [34].

Counterfeit manufacture may increase the risk of physical injury. Approximately 30% of car parts in the Near East and 80% in Africa are believed to be counterfeit, coming mainly from China, South Korea and India. Counterfeit car bonnets on sale in France have been described as acting like *"veritable guillotines" *in crash tests [21]. Counterfeit electrical goods carry a risk of fire and electric shock [53]; it has been estimated that 30-50% of electrical goods sold in the Far East are counterfeit, while in Africa estimates range from 25% to 80% [21]. There is some evidence linking the Italian mafia to the distribution of counterfeit electrical goods produced in China [54].

## The illegal drug trade

In 2005 the UN Office for Drug Control estimated that the international drug market is worth US$300-500 billion per year, representing about 0.9% of global GDP, or the equivalent of around three-quarters of the GDP of all sub-Saharan African countries combined. One estimate is that 40-50% of the income of organised crime comes from drug traffic, in opiates, cocaine, cannabis, or synthetics. The last two can be produced in many places, while opiates originate mainly in Asia's Golden Crescent (Afghanistan, Iran, Pakistan) and Golden Triangle (Burma, Laos, Vietnam and, formerly, Thailand), and in the Andes poppy production is nowadays supplementing the traditional coca [6].

Widespread demand for psychoactive drugs is mainly serviced through illegal supply in which organised crime is a major player: this is the main source of funds for many criminal groups, such as Albanian, Turkish and Russian mafia, Chinese Triads and the Japanese Yakuza. New players continue to enter the game: West Africa has now become a significant drug trafficking nexus. In addition to any direct involvement, organised criminals may exact *"taxes" *from those involved in the drug trade (sometimes in exchange for protection against other actors).

### Drug trafficking: source and transit countries

The illicit commerce that producers feed typically damages the communities through which it transits as well as the communities to which it is exported. This occurs because traffickers pay for goods and services en route in kind not in cash as much as they can: the recipient can realise the street value of the merchandise he receives, but it costs the payer only the purchase price plus the costs already invested in transporting it to that point, typically totalling much less than the street price. As local recipients offload their payments-in-kind into neighbouring communities in order to extract their cash, concentrations of users build up around drug transit points. This generates localised prevalence peaks of blood-borne hepatitis and HIV in places where heroin moves from one form of transport to another. The border points where it enters and leaves a country also develop high HIV rates through the same mechanism, as officials are paid to permit its transit: this explains why some of China's highest HIV prevalences are at Ruili on the China-Burma border and Yining on the Kazahk-China border, marking the entry and exit points of Golden Triangle heroin [55]. Land-based traffickers too suffer elevated rates of heroin use, for example Albanians, Bedouins and Turks [5]. Where drug users get into debt as a result of their addiction, other forms of harm may appear, such as sale of pre-pubescent girls to traffickers[56,57].

## Trafficking and smuggling human beings

Trafficking is defined by a supplementary protocol to the 2000 Convention against Transnational Organised Crime, as follows:

"Trafficking in persons shall mean the recruitment, transportation, transfer, harbouring or receipt of persons, by means of the threat or use by force or other forms of coercion, of abduction, of fraud, of deception, of the abuse of power or of a position of vulnerability or of the giving and receiving of payments or benefits to achieve the consent of a person having control over another person, for the purpose of exploitation. Exploitation shall include, at a minimum, the exploitation of the prostitution of others or other forms of sexual exploitation, forced labour or services, slavery or practices similar to slavery, servitude or the removal of organs."

Source: Protocol to Prevent, Suppress and Punish Trafficking In Persons, Especially Women and Children, Supplementing the United Nations Convention Against Transnational Organized Crime [58]

A review of data from multiple sources, including Interpol, non-governmental organizations, and the US Congress estimated that 700,000 to 2,000,000 people are trafficked each year, involving financial flows of $12-$32 million [6]. These figures include persons trafficked into a multiple and diverse forms of exploitation, including domestic labour, agricultural labour, begging, and sex work. Only the last of these carries a significant risk to public health, though workers may find themselves in conditions of unforeseen hardship. Some women volunteer for sex work abroad, for example many of the Thai women who sell sex in Japan [59]. Many more do not know what awaits them as they are recruited by deception, kidnapping or betrayal.

A US government report on all forms of human trafficking estimates that around 43% of trafficking is undertaken to supply women and girls for forced commercial sex [60]. There are frequent accounts of Asian women recruited as maids, nannies, and factory workers in the Middle East being forced to sell sex on arrival at their destination[61].

### The sex industry: trafficking and provision of adult women to provide sexual services

**Figure 2:**
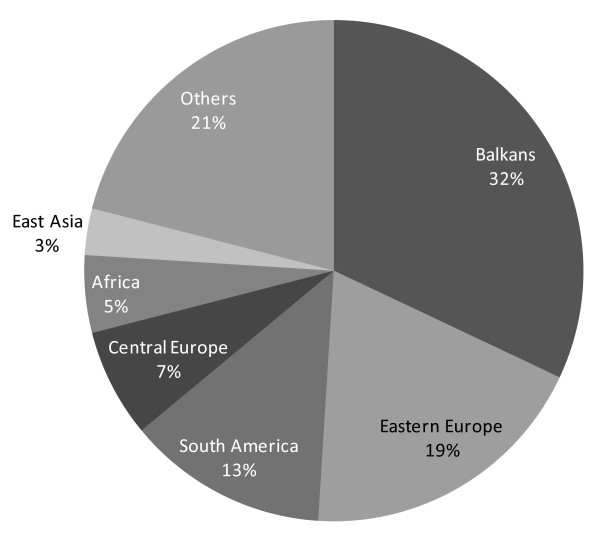
**Nationalities of trafficking victims detected in West and Central Europe 2005-2006 (%)**. Predominant source countries with regions: East Asia: Thailand, China, Vietnam and Cambodia; Africa: Nigeria; South America: Brazil; Eastern Europe: Russian Federation, Ukraine, Moldova, Bulgaria & Romania. Source: UNODC [73].

Commenting on a paper by Zimmerman et al [62] on the health problems facing women who had been trafficked, Beyrer has argued that the trafficking of women and girls into forced sex for commercial gain should be viewed as "*one of the most disturbing health issues of our time*" given the consequences for HIV/AIDS, sexually transmitted infections, and mental health [63], as well as being a grave abuse of human rights [58]. UNODC estimates that 140,000 people are trafficked for sexual purposes each year [64], a figure that is believed to be increasing rapidly [65]. Figure 2 shows the nationalities of those identified as having been trafficked in western and central Europe in 2005-6.

This trade is a major source of funds for organised criminals [10], who use existing networks established to move other commodities internationally [10]. For their *"owners"*, trafficked women and children are *"productive assets" *that can either be sold outright to final users (pimps or brothel owners) or to other merchants who sell them on along the supply chain to their end user who rents them out to perform sexual services in exchange for money. A share of the customer's payment may or may not be paid to the person rented out. Often women and girls caught up in organised crime are debt-bonded: a cash sum is paid to the families of girls/women who leave for *"jobs" *elsewhere, and those tied by the debt are told that they have to repay this amount (plus various other costs) in kind by sex work, often stretching over years and carrying high risks of health damage.

The rewards for those controlling the trade are impressive compared to other businesses: the repeated sale of the sexual services of a sexually attractive young person can generate substantial income over a period of months to years, whereas a shipment of drugs can only be converted into cash once. The costs of running a brothel are not high, other than the expense of avoiding formal police investigations, in locations when this activity is illegal; in many cases male police officers accept bribes in kind by being serviced without charge (unpublished focus group of former female sex workers, Kunming, China, 2001). Penalties for pimps tend to be both low and inadequately enforced [66], and trafficked women are attractive to criminals because they can cross borders legally and will do so willingly for as long as they believe that they are heading for work as a dancer, a model, a waitress or a maid.

Sometimes there is vertical integration within the mainstream entertainment sector: nightclubs and bars offer opportunities for laundering money from illegitimate enterprises as they are cash-based businesses [67], and this is thus a common secondary activity for organised criminals. Provision of sex services in these facilities is also used for bribes [66] or in gathering material for blackmail, as well as being a very productive cash cow in its own right. There is competition for the most profitable markets; while the sex trade in the Bali entertainment district was run by Javanese traffickers until a few years ago, now the Russian mafiya has reportedly taken over (personal communication, former Indonesian Human Rights Commission worker on sex trafficking, 2010).

Global revenues from trafficking in women were estimated by the Global Survival Network to be US$7 billion per annum [61], with hundreds of thousands (at a conservative estimate) of women and girls taken each year. An estimated $3.1 billion of this arises from selling women in Europe [64]. Yet penalties for human trafficking are absent, weak or unenforced in many countries [61].

Globalisation has diversified the opportunities available to this industry, with international crime networks exploiting the burgeoning demand for *"exotic" *prostitutes for whom premium rates can be charged [61]. Examples include Nigerian, Russian and Columbian women trafficked to Japan, Ukrainians to Bangkok and the Middle East, and Thais to Europe [5]. Ukrainians are very much in demand; the Ukrainian Parliament's Committee on Human Rights estimates that thousands are captive in brothels in Western Europe and Israel. [68]

Both unwilling victims and compliant recruits to sex work are at high risk of contracting HIV and TB [69]. Criminal gangs typically privilege profits over the protection of sex workers' health, and may demand client numbers or practices which cause significant physical injury to mucous membranes, facilitating both contracting and passing on infections. They often prevent the women they control from insisting on condom use, because prices and client satisfaction are higher for unprotected sex, and the women can be cheaply replaced when their health deteriorates enough to make them unattractive.

The communities into which prostituted women are sold and those to which they return both experience elevated rates of sexually transmitted diseases. Silverman et al conducted surveys among 287 Nepali [70] and 175 Indian trafficking victims [71]. They found HIV rates of 38% in the Nepalese sample, while 23% of the Indian sample was seropositive, with the risk rising by 3-4% for each additional month a girl was held in a brothel. They note that these figures are likely to be underestimates, as testing typically happens immediately after rescue, thus infections in the preceding few weeks will not register on antibody tests.

Unsurprisingly, trafficking has severe consequences for mental health [72]. For many recalcitrant victims, initiation into their new occupation involves breaking their will through rape [62,71,73,74]. Research on women trafficked by the Russian mafiya to the USA identifies drugging, starvation and murder threats as common means of enforcing the will of their captors [66,73]. Depression, anxiety, addiction and post-traumatic stress disorder are common among women who have been exploited in this way. Attempts to escape such a life may be more dangerous still for captive sex workers: for example, in 1998 two Russian women were thrown to their deaths from a balcony in Istanbul to motivate compliance from six other reluctant women, a trafficked woman was beheaded in Serbia for refusing to engage in sex work in 1997 [75], and a Moldovan woman who attempted escape was shot in the knees and left in the Egyptian desert to die by her Bedouin traffickers in 2002 [5].

Sex workers, whether trafficked or not, are subjected to elevated rates of violence. A US study of 68 women who had worked in the sex trade for at least 6 months, found that half had been physically assaulted by clients; overall one-sixth experienced such assaults several times a year. 23% of assaults caused fractures and two women had been beaten into a coma [76]. Similar studies from other parts of the world have found equally high rates of violent assault (Table 1), with the worst situation seen in the study which looked at a group of women all of whom entered prostitution by being trafficked [70]: all of that sample had been assaulted. Vulnerability to sexually transmitted infections, including HIV, is enhanced by violence against sex workers [77].

**Table 1:** Surveys of violence against female and transgendered sex workers

Study	**Hunter **[115]	**Farley et al **[116]					**Gupta et al **[74]
**Year**	**1993**	**1998**					**2009**

**Location**	**Oregon, USA**	**San Francisco, USA**	**Johannesburg & Capetown, South Africa**	**Northern Thailand**	**Istanbul, Turkey**	**Lusaka, Zambia**	**Mysore, India (trafficked women)**

Sample size	55	130	68	110	50	117	61

% reporting rape by pimps and/or clients	78% (average 49 rapes per woman per year	68%	57%	57%	50%	78%	100%

% reporting physical assault	84% (aggravated assault)	82%	66%	55%	80%	82%	No % given, but stated to be usual means for pimps to address refusal to service clients

% mutilated	27%	No data	No data	No data	No data	No data	No data

While attention has focused largely on harm to women trafficked into prostitution, the risk to women married to clients of sex workers is frequently overlooked. About 70% of female infertility is caused by sexually transmitted infections passed on by husbands or long-term partners [78], some of whom will have contracted them through buying sex. In some countries, female infertility is a reason for divorce. Wives so rejected may have no choice but to move into sex work themselves: this has been documented in Niger, Uganda and the Central African Republic [61]. A recent review by UNAIDS concluded that, in Cote d'Ivoire, Lesotho, Ghana, Benin, and Peru, the number of HIV infections among the partners of clients of female sex workers may exceed the number of infections among the clients themselves [79].

### Trafficking in children

Criminal groups are increasingly active in the traffic in children for sexual and labour exploitation [80], as the profile of profit and risk of this activity is so favourable in comparison with more traditional vice activities. Moving children around the world to satisfy shifting demand can earn millions of dollars [13]. It is believed that half of all trafficked females are under eighteen years of age [81]. The youngest girls are often in especially high demand for sex services and so are required to take large numbers of clients. Zalisko cites the case of a 15-year old girl from Eastern Europe forced by her pimps in the USA to service up to twenty men per day [66]. Gupta et al note that since purchase of younger girls is more expensive for brothel owners (almost double the cost in their Indian study site), they are keen to recoup their investment quickly by maximising income [74].

Opportunist criminals move in when children are unaccompanied and vulnerable. For instance, after Ceausescu's downfall, Romanian children were trafficked for use in pornography and supplied to paedophile rings [13]; child traffickers were among the earliest arrivals at the site of the Aceh tsunami [82-84]. Virgins are at increased risk of infection with HIV and other sexually transmitted infections as bleeding of a ruptured hymen facilitates infection; men purchasing this experience see no need to use a condom to protect themselves [85].

Younger girls may also be at greater risk of cervical ectopy from physical trauma, and thus of infection [74,86,87], especially if the sex is forced on the girl[85]. Silverman et al found 61% HIV seropositivity among Nepali girls trafficked before the age of 15 compared to the 38% rate found among their adult peers (an adjusted odds ratio of 3.7); this difference was attributed by the authors to the higher demand for very young girls [70].

### Smuggling of international migrants

Another profitable area for organised crime is the provision of smuggling services to migrants, defined by the other supplementary protocol to the 2000 convention as follows:

*"the procurement, in order to obtain, directly or indirectly, a financial or other material benefit, of the illegal entry of a person into a state party of which the person is not a national" *[88]

Despite paying large sums for their journey (one author quotes sums of US$15-20,000 for transport from Sri Lanka to Europe, and $40-50,000 from China to the USA via the snakehead system[6]) migrants may be transported under extremely hazardous conditions, with large numbers dying in transit. In some cases their deaths are inadvertent, reflecting neglect, but in others they are murder; in the 1990 s, people who purchased a transit from Albania to Italy were taken to open waters and thrown overboard to drown, so that the boats could return faster to load more paying passengers[8].

The illegal status of many of these migrants renders them vulnerable to exploitation on arrival at their destinations, leading them to work long hours in hazardous conditions, a situation illustrated graphically by the drowning of 18 Chinese migrant cockle pickers in the north of England in 2004, stranded by an incoming tide [89].

## Organ trafficking

The Protocol to Prevent, Suppress and Punish Trafficking in Persons, Especially Women and Children, which forms Annex II to the UN Convention, covers *"the removal of organs" *in addition to *"the exploitation of the prostitution of others" *and allied offences against natural justice. There is now a thriving market in illegal transplants [90]. The commonest nationalities of recipients are the Gulf States, Japan, Italy, Israel, the USA and Canada, using organs harvested from India, Pakistan, Turkey, Peru, Mexico, Romania and South Africa [91]. In addition to the ubiquitous shortage of donors, there are some country-specific factors, such as the strict criteria for brain death applied in Japan. The situation in Israel, where some interpreted Jewish law as prohibiting donation, although not receipt, has recently been addressed in a law that gives those who have signed donor cards higher priority if they require a transplant [92]. Organs Watch has identified trafficking in all of the twelve countries where it has investigated allegations, which are, in addition to the above, Argentina, Brazil, Cuba, Iran, Moldova, the Philippines, Russia and the West Bank. It notes the involvement of organised crime in some countries, such as Brazil. There have been relatively few arrests worldwide, a recent exception being a group of businessmen and officials in New Jersey in July 2009 [93].

Reports of criminal involvement in the organ trade almost always involve live donors who usually have consented to sell a kidney. However, this may be accompanied by coercion; Moldovan men who travel to Turkey to sell a kidney have found themselves forced into signing consent forms at gunpoint [91]. There are media reports of killings of street children in South America and of the children of impoverished parents in Eastern Europe [94], but the Bellagio Task Force found no evidence to substantiate this and Organ Watch has not been able to confirm or disprove these reports.

The recipients of organs sourced through criminal networks may be exposed to risk of disease, since those involved in the trade organised crime are unlikely to take an interest in screening donors for blood-borne viruses. An example is the theft of body parts from the recently deceased in New York. Between 2003 and 2005, unscrupulous medical supply companies took bone and other tissues from corpses at funeral homes, which were transplanted into an estimated 20,000 people [95]. Not only did many of the deceased die of metastatic cancers, sepsis or blood-borne infections (as was the case in almost half of the 48 corpses that the authorities were able to match to their medical records), but their bodies were dismembered under non-sterile conditions permitting cross-contamination, usually after the 15-hour time limit for tissue harvesting, and often several days post mortem. Screening processes were circumvented by fraudulent provision of pathogen-free blood from other bodies[96]. At least two resulting cases of hepatitis C have been documented [97] and one of syphilis; another known recipient almost died of septic shock, and one did die of fungal infection [96].

## What can be done?

Many of the issues discussed in this paper are the subject of existing international agreements but, as with the Convention against Transnational Organised Crime, any consideration of health-related matters is typically incidental.

The few exceptions are those issues where there is an obvious link to health. One is counterfeit medicines. In 1994 the World Health Assembly passed Resolution WHA47.13 requesting the Director-General to assist Member States in ensuring that available medicines were of good quality and in combating the use of counterfeit drugs. In 2006 the WHO set up the International Medical Products Anti-Counterfeiting Taskforce [29] to address the issue. The EU is extending the use of package tagging systems incorporating RFID or 2-dimensional data matrix barcode technology [27]. Another is the illegal trade in cigarettes, which is a subject of the World Health Organization's Framework Convention on Tobacco Control. A recent review identified successes by the authorities in Italy, Spain and the United Kingdom [98].

Some initiatives encouraged by these agreements have achieved a degree of success. In 2009 India introduced a scheme which pays whistleblowers for information on *"spurious, adulterated, misbranded and sub-standard drugs" *[99]. In 2000-1 the Nigerian President replaced the management of the National Agency for Food and Drug Administration and Control (NAFDAC) and gave the new managers greater powers [100] The new agency head strengthened import and export control, limiting legitimate entry and export to two seaports and two airports, and outlawing overland importation. Application of intensive controls, including a six-month closure of a major public market, reduced the percentage of fake drugs from an estimated high of 70% down to 20% by 2004, according to NAFDAC data [34], and to 16% by the beginning of 2006 [29]. However, this process has met with some resistance from those whose illicit profits were hit: in 2002 the policeman protecting the NAFDAC laboratory was cut to pieces with machetes, the head of NAFDAC survived an assassination attempt in 2003, and arsonists attacked its laboratories and headquarters in 2004[100].

Yet much more could be done. The pharmaceutical industry could facilitate action against counterfeiting of its products by making available the data collected on the scale and nature of counterfeiting by its Pharmaceutical Safety Institute (a consortium of 21 manufacturers) to researchers and enforcement agencies. Its extensive database is confidential, as its members' share prices are sensitive to information on the scale of counterfeit products[101]. They are instead, in the interests of protecting their profits, inclined to target measures against "*counterfeits" *to discredit and restrict access to legal generics and thus reduce access to essential medicines for the poor. Governments are sometimes yielding to pressure to enshrine this approach in law: the Uganda Anti-Counterfeit Bill takes this approach, in defining *"counterfeiting" *not as relating to incorrect or dangerous content of goods, but to its production without the authority of the intellectual property right attaching to that type of goods. This has the effect of rendering the government liable to prosecution if it has to use the emergency compulsory licensing provisions provided for under the TRIPS agreement to produce an essential antidote to an epidemic disease outside its patent (as used by the USA to produce non-patented ciprofloxacin for bulk distribution during the 2001 anthrax scare).

Other agreements have stimulated little action. Thus, the 1949 Convention for the Suppression of the Traffic in Persons and of the Exploitation of the Prostitution of Others criminalises pimping but its provisions are rarely enforced.

One problem is that there is considerable controversy about how to address some of these issues and there are often unintended consequences of seemingly logical responses. An example is drug prohibition. Efforts to combat the ill-effects of narcotics have stressed supply reduction over demand reduction or harm reduction, using punitive measures rather than measures rooted in public health and human rights [102]. Yet experience in 19^th ^century Europe suggests that prohibition of drugs encourages smuggling and the development of illicit markets [14]. Enforcement against drug traffickers may also be dangerous to those in the communities where they live; in Brazil, 15,000 people *"disappeared" *following contact with security forces during a clamp-down, and the number of deaths from *"resisting arrest" *more than tripled [103].

Measures to reduce supply often increase the profitability of growing, transporting and marketing recreational drugs [104], thus increasing the profits made by criminals. Buxton identifies the *"prohibition ratchet" *[14], which operates as follows. If supply shrinks, because of enforcement or other reasons, retail prices increase but volume is constrained, leaving a portion of the demand unsatisfied. The consequence is typically increased adulteration by retailers, increased crime by users to meet the higher cost of a stable habit (in economic terms addictive substances have inelastic demand, so increase in price does little to reduce use), and increased incentives to producers and traffickers. As well as the boosts in profits that this mechanism affords to traffickers, the level of adulteration is permanently affected. Each supply constriction reduces street purity, but without a return to previous levels when supply constraints ease and the wholesale price of pure drug falls. This happens because adulteration is a form of rationing of the active ingredient across all of the dealer's clientele during a market drought. With non-toxic adulterants, users adjust their psychological expectations to the new lower concentration of active ingredient and start to compensate by using higher quantities. When supply eases, most dealers continue to adulterate to the same proportion as during the drought, since buyers are not likely to complain and profits are multiplied for the dealer by doing so. When unexpectedly pure opiate supplies enter the market the usual consequence is a spate of overdoses, as users overestimate the amount needed to get high and thereby administer enough of the drug to cause their death.

The War on Drugs in its many forms may also alter users' behaviour to the detriment of public health. According to former heroin addicts interviewed in Yunnan in 2002, novice opium addicts soon switch to injecting heroin so the police cannot smell fumes from their activities; by 2002 HIV was infecting around half of local injectors according to local government statistics, due to sharing of scarce injecting equipment. In many countries, police stake out needle and syringe exchanges, or pharmacies which sell sterile syringes [105] and arrest users, thus forcing them to reuse injecting equipment which may be contaminated with pathogens, including blood-borne viruses. Fear of arrest can lead to rushed injection (increasing overdose risk), sharing of syringes (as being caught with a syringe can be grounds for arrest) and reluctance to use harm reduction services. Police crackdowns can displace drug users to areas where they cannot access services. There are also reports of extrajudicial execution of drug users, as in Thailand in 2003.

Over-incarceration of drug users in prison and pre-trial detention settings also creates breeding grounds for infectious disease, particularly HIV, blood-borne hepatitis and TB [106]. Injecting drugs in prison is particularly risky because the often copious drug supply is not associated with liberal provision of clean syringes or the means to sterilise used injecting equipment. Thus the organised criminal activity which supplies the demand for drugs in prisons is combined with misguided prevention measures to result in elevation of the risk of transmission of HIV and blood-borne hepatitis.

To the degree that it has had any success in reducing the involvement of organised crime in the narcotics trade, the *"war on drugs" *appears merely to have displaced the problem: some of the organised criminal groups facing increased costs have moved into counterfeiting of pharmaceuticals [25]. International experts on HIV are now calling for a halt to criminalisation of drug users and a move to evidence-based policy on dealing with the drug trade, through the Vienna Declaration. The Declaration lists, among the consequences of existing approaches to law enforcement *"HIV epidemics fuelled by the criminalisation of people who use illicit drugs and by prohibitions on the provision of sterile needles and opioid substitution treatment"*[107].

Another contentious issue is legalisation of prostitution, often promoted as a measure to protect public health. In Sweden, where buying sex is illegal but selling it is not, only 0.03% of the population sell sex, but Germany, with its legal prostitution sector, has 0.38% of its residents selling sex, more than twelve times as many [108]. Actually legalisation seems more aimed at protecting the health of clients, while increasing risks to the people who service them [108]; it has the effect of increasing the number of customers and the prices they can be charged, generating extra revenue to service operators.

Where the law incorporates HIV-certification and illegality of sex work for HIV+ women, as in Austria [109] risk will be shifted from clients on to sex workers if clients interpret HIV-free certification as meaning that condom use is unnecessary; this has been seen in practice [110]. It might be felt that the extra risk to sex workers is justified by the overall reduction in HIV at population level achieved by removing HIV+ women from selling sex legally, but actually it is hard to know whether this would be the outcome. Since up to half of HIV transmission occurs during the window period [111,112] it is by no means clear that the outcome of required HIV tests for legal sex workers will be a reduction in HIV incidence if it induces complacency which leads to an increase in commercial sex with a reduction in condom use. Thus the excellent protection for unprotected sex that customers believe is offered to them by legal sex establishments which enforce regular HIV testing on the providers may be illusory: each new client is exposed to the possibility that his partner may be in the highly contagious primary infection phase following infection in the previous few weeks, in which case he will be exposed to a high concentration of virus during the sexual act. This may be more likely to result in infection than unprotected intercourse with someone in the chronic asymptomatic phase of infection, when viral load is comparatively low in most cases.

In any case many HIV+ sex workers merely shift into the illegal sector, which is not removed by legalisation; in fact the legalisation of sex work accompanied by regulation implies the existence of an illegal sex sector, populated by those who cannot meet the regulatory requirements, usually because they are too young, too diseased or are illegal migrants. The unregulated sector in Victoria, Australia, doubled in size after legalisation in 1994, and the safety monitoring schemes for which part of the Victorian brothel licence fees were supposed to be used have never materialised [113]. Decriminalisation of sex work thus offers the best option to reduce its public health and human rights risks. Under such arrangements, there are no grounds for police to harass those who sell their bodies, no illicit adult vendors, and at the same time no legitimisation of the procurers who may threaten sex workers' health, safety and liberty, and the health of the communities they operate in.

The need for concerted international action, the existence of strong vested interests opposing it (and in particular the existence of corruption), and the controversial nature of many of the putative solutions will inevitably make it difficult to address the public health consequences of organised crime. However, it would be wrong for the public health community simply to place it in a *"too difficult" *category. In recent years this community has engaged effectively with many different sectors to begin to tackle the broad determinants of health. We have argued that organised crime is one more of these determinants to be addressed.

Clearly it is not appropriate for public health to take the lead on this issue. The criminal justice system will rightly maintain primacy. But we do believe that a public health approach may have something to offer.

First, it recognises the importance of looking upstream, avoiding what has long been termed *"victim blaming" *in which existing law enforcement measures often criminalise the victims, such as those who have been trafficked, or vulnerable people who have developed addictions. They are often much easier to identify than those who control the business, and are unable to evade the consequences through bribery or intimidation.

Second, it emphasises the importance of evidence of effectiveness. Unfortunately, there is still limited research to draw on; the Campbell Collaboration http://www.campbellcollaboration.org does now contain systematic reviews of interventions to tackle crime, but so far most address primarily micro-level issues. Rigorous and comprehensive evaluation is especially important in this area given the evidence reviewed above showing the scope for unintended consequences.

Third, it has long recognised the importance of context. Organised crime is more common in countries when the rule of law is weak. An absence of high-level political interference, strong private sector governance and regulation, an effective judicial system, and an independent and honest judiciary all deter corrupt behaviour and weaken criminal networks [114]. Research on illicit drug use in both recipient and supplier countries identifies how *"the capacity of the state to maintain a viable and legitimate presence in local communities determined the extent to which drug-related activities developed and consolidated at local level" *[14]. Consequently, it is necessary to take account of the quality of governance in implementing any measures.

Fourth, a public health approach stresses the importance of inter-sectoral collaboration. Addressing the major threats to health today requires inputs from many sides. Organised crime is no exception. Yet, too often the relevant experts exist within silos, rarely seeing the need to engage with each other. The public health community can play a convening role, helping to break down these barriers.

To conclude, the tentacles of organised crime have huge reach, as demonstrated above, and may adversely impact the health of many millions of people around the globe. Through fear induced by brutality and corruption, the perpetrators enjoy not only a high level of impunity but also extraordinary invisibility. We hope that, by bringing together a wide range of evidence from disparate areas of research, we have begun to make the case for a joined-up, evidence-based approach to addressing the global health consequences of organised crime.

## Competing interests

The authors declare that they have no competing interests.

## Authors' contributions

MM conceived of the study, and edited the manuscript for publication. LR reviewed the literature and drafted the paper. Both authors read and approved the final manuscript.
